# Clinical Pharmacokinetics of Penicillins, Cephalosporins and Aminoglycosides in the Neonate: A Review

**DOI:** 10.3390/ph3082568

**Published:** 2010-08-12

**Authors:** Gian Maria Pacifici

**Affiliations:** Section of Pharmacology, Department of Neurosciences, Medical School, University of Pisa, Via Roma 55, 56126 Pisa, Italy; E-Mail: pacifici@biomed.unipi.it; Tel.: +39-050-22-18-721; Fax: +39-050-22-18-717

**Keywords:** penicillins, cephalosporins, aminoglycosides, pharmacokinetics, neonate

## Abstract

Bacterial infections are common in the neonates and are a major cause of morbidity and mortality. Sixty percent of preterm infants admitted to neonatal intensive care units received at least one antibiotic during the first week of life. Penicillins, aminoglycosides and cephalosporins comprised 53, 43 and 16%, respectively. Kinetic parameters such as the half-life (t_1/2_), clearance (Cl), and volume of distribution (Vd) change with development, so the kinetics of penicillins, cephalosporins and aminoglycosides need to be studied in order to optimise therapy with these drugs. The aim of this study is to review the pharmacokinetics of penicillins, cephalosporins and aminoglycosides in the neonate in a single article in order to provide a critical analysis of the literature and thus provide a useful tool in the hands of physicians. The bibliographic search was performed electronically using PubMed, as the search engine, until February 2^nd^, 2010. Medline search terms were as follows: pharmacokinetics AND (penicillins OR cephalosporins OR aminoglycosides) AND infant, newborn, limiting to humans. Penicillins, cephalosporins and aminoglycosides are fairly water soluble and are mainly eliminated by the kidneys. The maturation of the kidneys governs the pharmacokinetics of penicillins, cephalosporins and aminoglycosides in the neonate. The renal excretory function is reduced in preterms compared to term infants and Cl of these drugs is reduced in premature infants. Gestational and postnatal ages are important factors in the maturation of the neonate and, as these ages proceed, Cl of penicillins, cephalosporins and aminoglycosides increases. Cl and t_1/2_ are influenced by development and this must be taken into consideration when planning a dosage regimen with these drugs. More pharmacokinetic studies are required to ensure that the dose recommended for the treatment of sepsis in the neonate is evidence based.

## 1. Introduction

Over 60% of the preterm neonates admitted to the neonatal intensive care units received at least one antibiotic during the first week of life, of which penicillins, aminoglycosides and cephalosporins comprised 53, 43 and 16%, respectively [1].

Sepsis in neonates has an estimated mortality of 10–20%, with sequelae in 25–30% of the infants who survived [2]. Immaturity of humoral, cellular and myeloid cell line immunity places the neonate at higher risk for infection than older infants and children [3]. The physiological conditions of neonates are different from those of adults. Neonates have a larger extracellular fluid volume [4] they also have immature liver and kidney functions as well as higher plasma concentrations of bilirubin and non-esterified fatty acids [5]. The water content is larger in preterm than in term infants [6] and penicillins, cephalosporins and aminoglycosides are fairly water soluble and are distributed in larger volume in preterm than term infants. These antibiotics are mainly eliminated by the kidneys and their renal glomerular filtration and tubular secretion are reduced in the neonate [7]. The reduced renal excretory function affects the disposition of penicillins, cephalosporins and aminoglycosides and their clearance (Cl) is reduced in newborn infants compared to children. The volume of distribution (Vd) of penicillins, cephalosporins and aminoglycosides tends to be larger in the neonate than in the adult because of the larger water body content in the neonate.

The extensive use of antibiotics in the neonate requires that their pharmacokinetics should be studied. The aim of this article is to provide the review of the literature on the pharmacokinetics of penicillins, cephalosporins and aminoglycosides in the infant in a single article in order to provide a tool that can be useful in the hands of physicians. This note also serves as an updated source of the literature on the pharmacokinetics of the penicillins, cephalosporins and aminoglycosides in the newborn infant.

## 2. Bibliographic Search

The bibliographic search was performed electronically using PubMed, as the search engine, until February 2^nd^, 2010. Medline search terms were as follows: pharmacokinetics AND (penicillins OR cephalosporins OR aminoglycosides) AND infant, newborn, limiting to humans. In addition, the book *Neofax: a Manual of Drugs Used in the Neonatal Care* by Young and Mangum [8] was consulted. The finding of the bibliographic search gave rise to 94 original articles, 11 review articles and two book chapters. The publication years of this subject matter ranged from 1961 to 2010. The references were “copied” from PubMed, “pasted” to the manuscript and edited according the style of the journal *Pharmaceuticals*.

## 3. Results

Table 1, Table 3 and Table 5 report the number of retrieved studies and the number of drugs evaluated for penicillins, cephalosporins and aminoglycosides, respectively. The pharmacokinetic parameters are clustered in three tables. Table 2 summarizes the pharmacokinetic results of penicillins, Table 4 shows the data relative to cephalosporins and the data relative to aminoglycosides are given in Table 6. The p-value refers to the data in the two rows above of Table 2, Table 4 and Table 6.

## 4. Penicillins

**Table 1:** Number of retrieved studies and number of drugs evaluated.

Drugs	Number of retrieved studies	Number of drugs evaluated	Overall number of neonates studied
Benzyl penicillin	3	1	67
Ampicillin	3	1	69
Amoxicillin	5	1	233
Flucloxacillin	3	1	75
Azlocillin	2	1	81
Mezlocillin	2	1	100
Ticarcillin	3	1	90
Total	21	7	715

### 4.1. Benzyl penicillin (penicillin G; see Table 2)

McKraken *et al.* [9], Metsvaht *et al.* [10] and Muller *et al.* [11] reported a study on the pharmacokinetics of benzyl penicillin in the neonate. Little is known about the gestational age; the half-life (t_1/2_) decreased from 3.2 h in low birth weight in the first week of life to 1.4 in week 3 [9]. In very low birth weight, t_1/2_ is 4.6 h. The Cl ranged from 1.2 to 1.7 mL/min/kg, Vd ranged between 0.41 and 0.65 L/kg, and t1/2 ranged from 1.4 to 4.6. The daily dose of benzyl penicillin ranged from 10-30 mg/kg [9] and from 15 to 30 mg/kg twice a day [10]. Young and Mangum [8] suggested a dose of 45 to 60 mg/kg for treatment of meningitis and a dose of 15 to 30 mg/kg for the treatment of bacteraemia. When the postmenstrual age ranged from ≤ 29 to ≤ 44 weeks, benzyl penicillin should be administered every 8 or 12 h, when the postmenstrual age ≥ 45 weeks the drug should be administered every 6 h.

### 4.2. Broad spectrum penicillins

#### 4.2.1. Ampicillin (see Table 2)

Three articles on the pharmacokinetics of ampicillin in the neonates have been reported [12,13,14]. In infants with a gestational age >37 weeks, t_1/2_ ranged from 2.2 to 3.9 h whereas in infants with a gestational age < 37 weeks t_1/2_ was 4.0 h. No data are available relative to Cl and Vd. Axline *et al.* [15] studied the effect of postnatal age on t_1/2_ of ampicillin. When the postnatal age ranged from 2 to 7, from 8 to 14, from 15 to 30 and from 61 to 68 days t_1/2_ was 4.0, 2.8, 1.7 and 1.6 h, respectively.

Young and Mangum [8] suggested a dose of 25 to 50 mg/kg and 100 mg/kg for the treatment of meningitis. When the postmenstrual age ranged from ≤29 to ≤44 weeks ampicillin should be administered every 8 or 12 h. When the postmenstrual age ≥ 45, ampicillin should be administered every 6 h.

**Table 2:** Pharmacokinetic parameters of benzylpenicillin. Figures are the mean or range.

Gestational age (weeks)	Daily dose (mg/kg)	Cl (mL/min/kg)	Vd (L/kg)	t_1/2_ (h)	Peak conc. (µg/mL)	Trough conc. (µg/mL)	Ref.
**Benzyl (penicillin G)**							
na	10–30 IM	na	na	3.2	22–36	na	[9]
na	10 IM	na	na	1.4	na	na	
<28	30 × 2	1.2	0.41	3.8	146	7	[10]
<28	15 × 2	1.5	0.65	4.6	59	3	
<32	30 × 2	1.7	0.50	3.9	na	na	[11]
**Ampicillin**							
>37	50–100 infusion	na	na	2.2	na	na	[12]
>37	50–100 ×2 IM	na	na	3.9	36–257	na	[14]
<37	50–100 ×2 IM	5.5	na	5.5	78–293	na	
na	50 ×3 IM	na	na	1.6	58	na	[15]
**Amoxicillin**							
<32	25 × 2	1.0	0.67	6.7	54	16	[17]
≤32	50 × 2	1.3	0.68	na	74	na	[16]
25–42	30	1.6	0.65	5.2	101	21	[18]
26–41	50–100	3.0	0.66	3.0	na	na	[20]
>37	50	na	na	na	38	13	[21]
<37	50	na	na	na	59	19	
**Flucloxacillin**							
26–42	25–50 × 3	3.0	0.54	2.6	38–114	na	[22]
33–41	50	0.7	0.28	4.6	na	na	[23]
na	30 × 3	2.0*	0.45*	2.6*	15–70*	na	[19]
**Azlocillin**							
<37	50 × 2 IM or IV	na	0.34	4.4	122	20	[24]
37–42	50 × 2 IM/IV	na	0.30	2.6	128	27	
37–42	100 × 2 IM or IV	na	0.30	2.6	260	17	
<37	50	3.7	0.33	2.5	na	na	[25]
37–42	50	4.9	0.32	2.6	na	na	
**Mezlocillin**							
26–40	75 IV or IM	1.3	0.38	3.7	231	43	[26]
<38	See text	9.3(mL/min per 1.73 m^2^)	0.34	4.5	252	72	[27]
38–42		12.3(mL/min per 1.73 m^2^)	0.37	3.0	272	34	
**Ticarcillin**							
31	75 × 2	na	na	4.5	183	43	[28]
34–36	83 × 3	na	0.34	4.2	279	64	[29]
>37	100 × 3	na	0.27	2.7	402	56	
30	75 × 2	0.78	0.26	4.2	976	42	[30]

na *=* not available. IM *=* intramuscular.

#### 4.2.2. Amoxicillin (see Table 2)

When amoxicillin was administered in association with gentamicin there was a decrease of amoxicillin Cl of 25% [16]. Charles *et al.* [16] found a prolongation of t_1/2_ and an increase of Vd when amoxicillin was co-administered with gentamicin in preterm infants. Huisman-de Boer *et al.* [17] and Pullen *et al.* [18] studied the pharmacokinetics of amoxicillin in neonates. Huisman-de Boer *et al.* [17] recommend a dose of 15 mg/kg every 8 h for neonates with gestational age < 34 weeks and 20 mg/kg every 8 h when the gestational age >34 weeks. Pullen *et al.* [18] suggested that preterm infants with gestational age < 32 weeks in the first week of life receive a maximum amoxicillin dose of 25 mg/kg every 12 h. Cardiopulmonary bypass decreased the mean plasma concentration by 36%, from 73 to 47 µg/mL [19]. t_1/2_ ranged from 3.0 to 6.7, Vd ranged from 0.65 to 0.68 L/kg and Cl ranged from 1.0 to 3.0 mL/min/kg. In the studies by Huisman-de Boer *et al.* [17] and Pullen *et al.* [18] Cl and Vd were found to increase with gestational age. Preterm infants have a longer t_1/2_ (6.7 h) than term neonates (3.0 h).

### 4.3. Penicillinase-resistant penicillins

#### 4.3.1. Flucloxacillin (see Table 2)

The dose of flucloxacillin ranged from 30 mg/kg thrice daily [19] to 50 mg/kg every 8 h [22]. Pullen *et al.* [22] observed that a dose of 50 mg/kg thrice daily did not result in effective plasma concentrations for the treatment of *Staphylococcus aureus*. They suggested using 25 mg/kg every 6 h for all neonates. Cardiopulmonary bypass decreased the plasma concentration of flucloxacillin by 40% [19]. Cl ranged from 0.7 to 3.0 mL/min/kg, Vd ranged from 0.28 to 0.54 L/kg and t_1/2_ ranged from 2.6 to 4.6 h. A small study of nine neonates suggested that t_1/2_ was related to gestational age [23].

### 4.4. Antipseudomonal penicillins

#### 4.4.1. Alzocillin (see Table 2)

Sitka *et al.* [24] suggested a dose of 50 mg/kg of azlocillin for preterm neonates in the first 7 days of life and 100 mg/kg every 12 h in full term neonates in the first 7 days of life. Another study [25] did not find difference in t_1/2_ in preterm and term infants. Cl ranged from 3.7 to 4.9 mL/min, Vd ranged from 0.30 to 0.34 L/kg and t_1/2_ ranged from 2.5 to 4.4 h.

#### 4.4.2. Mezlocillin (see Table 2)

The mezlocillin serum concentrations were influenced by the co-administration with an aminoglycoside [26]. Cl of mezlocillin increased with gestational age and was negatively influenced by aminoglycoside co-administration. There was no relationship between Vd and body weight. Janicke *et al.* [26] suggested a dose of 75 mg/kg every 8-12 h. Another study [27] recommended a dose of 75 mg/kg every 12 h to preterm infants in the first week of life and 75 mg/kg every 8 h for preterm and term infants with postnatal age >7. In one study, Cl was 1.3 mL/min/kg [26] and in another study [27], Cl ranged from 9.3 to 12.3 mL/min per 1.73 m^2^. Vd ranged from 0.34 to 0.38 L/kg and t_1/2_ ranged from 3.0 to 4.5 h.

#### 4.4.3. Ticarcillin (see Table 2)

Ticarcillin was administered in combination with clavulanic acid [29,30]. The ratios of ticarcillin:clavulanic acid was 15:1 [28] and 30:1 [29]. Another study used a 25:1 ratio of ticarcillin:clavulanic acid [30]. The dose of ticarcillin was 75 mg/kg every 12 h [28], 83 or 100 mg/kg thrice daily [29] and 75 mg/kg twice a day [30]. Vd ranged from 0.26 to 0.34 L/kg and t_1/2_ ranged between 2.7 and 4.5 h. Young and Mangum [8] suggested 75 or 100 mg/kg every 8 or 12 h up to a postmenstrual age of ≤44 weeks and every 6 h when the postmenstrual age was >45 weeks.

The findings more relevant from a clinical point of view have reported hereafter. Penicillins are rapidly eliminated mostly by renal route and their toxicity is limited. The kinetic parameters, including the peak concentration, range over a wide interval. Penicillins are mostly used to treat infections sustained by Gram-positive bacteria and are a key components in neonatal intensive care units. Gestational and postnatal ages influence Cl and t_1/2_ of penicillins. The minimal toxicity associated with penicillins in comparison with other antibiotics such as aminoglycosides and second- and third-generation cephalosporins is probably one of the reasons why there have been few studies on the kinetics of penicillin in the neonate. There is a considerable dose variation for both benzylpenicillin and flucloxacillin and, to a lesser extent, for ampicillin and amoxicillin.

## 5. Cephalosporins

**Table 3:** Number of retrieved studies and number of drugs evaluated.

Drug	Number of retrieved studies	Number of drugs evaluated	Overall number of neonates studied
Cefazolin	1	1	11
Cefoxitin	1	1	15
Cefuroxime	1	1	104
Cefotaxime	7	1	130
Ceftazidime	6	1	283
Ceftriazone	5	1	199
Cefoperazone	4	1	75
Ceftizoxime	2	1	102
Cefepime	3	1	93
Total	30	9	1012

### 5.1. First Generation Cephalosporins

#### 5.1.1. Cefazolin (see Table 4)

Deguchi *et al.* [31] studied the pharmacokinetics of cefazolin; there was a marked interindividual variability in Vd that ranged from 0.21 to 0.37 L/kg. The unbound fraction of cefazolin in neonatal plasma ranged from 0.22 to 0.83. There was a correlation (r *=* 0.936; p < 0.001) between cefazolin Vd and the plasma unbound fraction of this drug.

Young and Mangum [8] suggested administering 25 mg/kg cefazolin every 8 to 12 h when the postmenstrual age ranged from ≤29 to ≤44 weeks. When the postmenstrual age is ≥45 weeks the interval between doses should be 6 h.

**Table 4:** Pharmacokinetic parameters of cephalosporins. Figures are the mean or range.

Gestational age (weeks)	Daily dose (mg/kg)	Cl (mL/min/kg)	Vd (L/kg)	t_1/2_ (h)	Peak conc. (µg/mL)	Trough conc. (µg/mL)	Ref.
**Cefazolin**							
35	30	0.80	0.28	na	na	na	[31]
**Cefoxitin**							
36	30 × 3	4.5	0.50	1.4	69	na	[32]
**Cefuroxime**							
na	10 × 3	na	na	4.6	24	6.4	[33]
**Cefotaxime**							
Preterm	Note A	1.37(mL/min)	0.61	5.7	na	na	[34]
Term		4.45(mL/min)	0.69	2.0	---	---	
p	---	<0.05	NS	3.1	---	---	
Preterm	50	23.0(mL/min/1.73 m^2^)	0.51	4.6	116	34	[35]
Term	50	49.3(mL/min/1.73 m^2^)	0.44	3.4	133	38	
P	---	<0.001	<0.01	<0.01	NS	NS	
30	50 × 2	0.50	0.42	4.6	na	na	[37]
38	50 × 2	1.07	0.28	4	na	na	
p	---	NS	<0.05	NS	---	---	
<32	25 × 2	1.08	0.34	3.5	74	11	[36]
>37	50	2.33	0.36	2.0	68	22	
p	---	<0.005	NS	<0.001	NS	<0.05	
**Ceftazidime**							
35	50	na	na	4.7	109	12	[41]
37	50 IM	na	na	3.8	53	9.0	
p	---	---	---	<0.05	<0.001	<0.05	
≤32	Note A	0.98	0.53	6.7	111	41	[42]
≥38		1.42	0.48	4.2	102	29	
p		P < 0.05	NS	NS	NS	<0.05	
25–42	25 × 2	0.84	0.46	7.3	77	16	[43]
	25 × 2 IM	0.63	0.40	14.2	56	20	
p	---	NS	NS	<0.01	<0.02	NS	
31 ^a^	50 × 2	0.68	0.36	6.3	na	na	[44]
29 ^b^	50 × 2	0.46	0.36	9.4	na	na	
p	---	<0.05	NS	<0.05	---	---	
29 ^c^	25 × 2	0.51	0.36	8.7	na	na	[45]
29 ^d^	25 × 2	0.69	0.29	5.0	na	na	
p	---	<0.05	<0.005	<0.005	---	---	
29	25	0.46	0.32	8.1	na	13.1	[46]
29	25 × 2	0.41	0.30	7.1	na	42.0	
p	---	NS	NS	NS	NS	<0.001	
**Ceftriazone**							
na ^a^	50	0.37	0.45	16.2	124	34	[49]
na ^b^	Note A	0.70	0.57	10.4	188	23	
p	---	0.02	NS	0.001	0.04	NS	
32.4	50	0.28	0.33	15.4	153	54	[50]
	50 IM	0.28	0.32	15.8	120	54	
p	---	NS	NS	NS	NS	NS	
5 term neonates and 25 infants 6 weeks to 2 years old	50	0.85	0.38	5.2	230	na	[51]
	75	0.93	0.39	5.4	295	na	
p	---	NS	NS	NS	<0.05	---	
**Cefoperazone**							
na	25 IM	na	0.24	3.9	66.3	3.7	[56]
na	25	na	0.20	4.0	93.1	5.2	
p	---	---	NS	NS	<0.05	NS	
na	12.5 IM	na	0.27	4.4	41.2	1.5	
na	12.5	na	0.20	4.3	45	3	
p	NS	NS	NS	NS	NS	NS	
32–36	50	0.60	0.12	5.5	136	na	[57]
	250	0.58	0.11	2.8	720	na	
p	---	NS	NS	NS	<0.001	---	
27–32	50	na	0.44	8.9	159	17	[59]
≥37	50	na	0.45	7.2	109	13	
p	---	---	NS	NS	<0.05	NS	
**Ceftizoxime**							
36 ^g^	Note D	0.68	0.37	7.2	na	na	[60]
na ^h^		2.4	0.44	2.4	na	na	
p	---	<0.05	NS	<0.05	---	---	
30	25 × 2	25.2	0.32	9.7	90	27	[61]
34		28.5	0.34	8.4			
p	---	NS	NS	NS	---	---	
**Cefepime**							
30	50 × 2	1.1	0.43	4.9	89	18	[62]
na ^i^	50 × 3	2.7	0.43	1.9	184	6	[63]
31	50 × 2	1.2	0.41	4.3	120	53	[64]

na *=* not available. IM *=* intramuscular. NS *=* not significant. Note A: Cefotaxime dose was 25 mg/kg and 50 mg/kg in patients with meningitis. Doses were administered every 12 h in neonates less than one week of age, and every 8 h in patients 1 to 4 weeks of postnatal age. ^a^ Controls. ^b^ Treated with indomethacin. ^c^ 3 days old. ^d^ 10 days old. na *=* not available. Note B: Dose of ceftazidime was 50 mg/kg every 12 h for neonates in the first week of life and every 8 h for older infants. Postnatal age of 1.7 days ^e^ and 17.4 days ^f^. Note C: 3 patients received 50 mg/kg; 4 patients received 100 mg/kg and 1 patient received 144 mg/kg ceftriaxone intravenously. Body weight 2,600 ^g^ and 4,600 ^h^. ^i^ Postnatal age ranged from 2 to 6 months. Note D: 25 patients received 25 mg/kg and 27 patients received 50 mg/kg.

### 5.2. Second generation cephalosporins

#### 5.2.1. Cefoxitin (see Table 4)

The pharmacokinetics of cefoxitin was studied by Regazzi *et al.* [32]. The half-life negatively correlated with postnatal age (r *=* −0.58; p < 0.05). Young and Mangum [8] suggested administering 25–33 mg/kg cefoxitin every 8 to 12 h when the postmenstrual age ranged from ≤29 to ≤44 weeks. When the post-menstrual age is ≥45 weeks the interval between doses should be 6 h.

#### 5.2.2. Cefuroxime (see Table 4)

Renlund and Pettay [33] studied the pharmacokinetics of cefuroxime in 104 neonates. The serum concentration of cefuroxime decreased with the body weight from 25.6 µg/mL ( <1 kg body weight) to 19.5 µg/mL (>4 kg body weight). t_1/2_ had a consistent behaviour and decreased from 5.6 h (2.83 kg body weight) to 4.0 h (3.83 kg body weight). Cefuroxime did not accumulate over a period of 8 days and this drug was excreted in the urine for over 70%. 

#### 5.2.3. Cefotaxime (see Table 4)

Four original studies on the pharmacokinetics of cefotaxime compare the kinetic parameters of this drug in preterm and term infants [34,35,36,37]. The gestational age ranged from 28 to 33 weeks in preterm infants and between 37 and 38 in term infants. Cl is higher in term than preterm infants and t_1/2_ is longer in preterm than term infants. Vd, peak and trough concentrations were not different in preterm and term infants. Gouyon *et al.* [38] observed that t_1/2_ of cefotaxime negatively correlates with gestational age (r *=* −0.8954; p < 0.01) and with body weight (r *=* −0.8500; p < 0.01). In contrast, Cl positively correlated with gestational age (r *=* 0.7280; p < 0.02) and with body weight (r *=* 0.8667; p < 0.02).

Cefotaxime is converted into desacetylcefotaxime in the neonate and the peak concentration of desacetylcefotaxime is about one tenth of that of cefotaxime [38,39,40]. After 50 mg/kg cefotaxime, 50% to 60% of the dose is excreted unchanged in the urine and about 20% is excreted as desacetylcefotaxime [39]. The renal Cl of cefotaxime is quantitatively more important than the metabolic Cl of this drug.

Young and Mangum [8] suggested a cefotaxime dose of 50 mg/kg every 12 or 8 h when the postmenstrual age ranged from ≤29 to ≤44 weeks. when the postmenstrual age was ≥45 weeks, cefotaxime should be administerd every 6 h. 

#### 5.2.4. Ceftazidime (see Table 4)

Boccazzi *et al.* [41] described the pharmacokinetics of ceftazidime after intravenous and oral administration. Peak plasma concentration was doubled after intravenous compared with oral concentration.

McCracken *et al.* [42] described the pharmacokinetics of ceftazidime in two groups of neonates with a gestational age of ≤32 and ≥38 weeks. There was a considerable variation in the concentration of ceftazidime. Cl increased with the gestational age whereas t_1/2_ and the trough concentrations decreased with the gestational age.

Cl of ceftazidime increased with postnatal age (r *=* 0.7035; p < 0.0001) and an opposite trend was observed with t_1/2_ and the postnatal age [r *=* −0.4951; p < 0.001; 43]. In the six infants examined, ceftazidime penetrated into the oropharyngeal secretion at concentrations equal to the MIC_90_ for Pseudomonas aeruginosa. The urine ceftazidime concentration was measured in 23 infants and it varied from 192 to 6,028 µg/mL. There was a dramatic effect of gestational age on the excretion of ceftazidime in the urine and in the serum Cl.

Prenatal exposure to indomethacin results in significantly lower GFR and ceftazidime Cl [44]. Cl of ceftazidime was 0.46 mL/min/kg (No *=* 23) in neonates who were exposed prenatally to indomethacin and 0.68 mL/min/mg (No *=* 84) in infants who were not exposed to indomethacin (p < 0.05). Cl of ceftazidime correlated with gestational age (r *=* 0.83; p < 0.001) whereas t_1/2_ had an opposite trend (r *=* −0.54; p < 0.001; 44). The positive relationship between Cl of ceftazidime and Cl of inulin (r *=* 0.73; p < 0.001) indicates that glomerular filtration has an important effect on the Cl of ceftazidime. However, the variability in Cl of ceftazidime exceeds Cl of inulin; this indicates that ceftazidime is not eliminated by glomerular filtration alone. Van den Anker *et al.* [44] propose that tubular reabsorption or tubular secretion is involved in the renal handling of ceftazidime in neonates. Cl of ceftazidime correlated with the reciprocal of the serum concentration of creatinine (r *=* 0.72; p < 0.001) suggesting that this compound may interfere with the renal clearance of ceftazidime.

Ceftazidime Cl increases from day 3 to day 10 of life [45]. Such an increase was due to the increase of GFR. The inulin Cl is 0.72 (day 3) and 0.91 mL/min (day 10) (p < 0.05). Cl of ceftazidime correlated with GFR (r *=* 0.81; p < 0.001). Such a correlation indicates the important role of GFR in the ceftazidime Cl. Vd of ceftazidime decreases between from day 3 to day 10 of life. During the first week of life there is a significant decrease of the extracellular water and ceftazidime is mainly distributed into the extracellular water component and the decrease of extracellular water may cause the decrease of ceftazidime Vd in the period 3 to 10 days of life. Postnatal exposure to indomethacin prevents the pharmacokinetic modification seen from the day 3 to 10 of life. This may be explained by the dependence of postnatal changes in extracellular water on renal function [45] and the impairment of GFR with the use of indomethacin.

Once-daily *versus* twice-daily administration of ceftazidime was studied by van den Anker *et al.* [46]. After 25 mg/kg twice-daily, the trough concentration of ceftazidime is too high (42.0 µg/mL) whereas the trough concentration of ceftazidime after once-daily dosing is 13.1 µg/mL and is greater than major neonatal pathogens MIC_99_ such as Streptococcus agalactiae and *Escherichia coli* (MIC_99_ < 0.25 µg/mL); [47,48]. Therefore, these authors suggested that once-daily 25 mg/kg ceftazidime is the appropriate therapeutic schedule of ceftazidime in the neonate. Cl ranged from 0.41 to 1.42 mL/min/kg, Vd ranged between 0.29 and 0.53 L/kg and t_1/2_ ranged from 3.8 to 14.2 h.

Young and Mangum [8] suggested administrating 30 mg/kg ceftazidime every 8 or 12 h according to the postmenstrual age ≤29 weeks and ≤44 weeks. When the postmenstrual age is ≥45 weeks, ceftazidime should be administered every 8 h.

#### 5.2.5. Ceftriaxone (see Table 4)

Martin *et al.* [49] studied the kinetics of ceftriaxone in 12 neonates. Four neonates received 50 mg/kg ceftriaxone whereas eight received a dose of ceftriaxone ranging from 50 to 144 mg/kg (see note A in Table 4). The gestational age as well was different in the two groups; it was 1.7 days in the first group and 17.4 days in the second group. Cl was greater in the former group, whereas t_1/2_ had an opposite trend. Such a difference could be due to the different development stage of neonates in the two groups. The peak concentration was greater in the neonates of the second group that received a higher dose of ceftriaxone. The intravenous and intramuscular administration of ceftriaxone yielded similar kinetic parameters [50]. Steele *et al.* [51] observed that the peak concentration of ceftriaxone was higher after 75 than after 50 mg/kg. Cl ranged from 0.28 to 0.93 mL/min/kg, Vd ranged between 0.32 and 0.57 L/kg and t_1/2_ ranged from 5.2 and 16.2 h. Young and Mangum [8] suggested administrating 50 mg/kg every 24 h. For treatment of meningitis give 100 mg/kg loading dose, then 80 mg/kg once daily.

Ceftriaxone should not be administered to neonates as it displaces bilirubin from albumin binding sites resulting in higher free bilirubin serum concentration [52,53]. Even more dangerous is the interaction of ceftriaxone with calcium. Such an interaction yields precipitation of calcium which resulted in serious adverse effect [54,55]. A particular serious effect is the precipitation of calcium in the lungs and death.

#### 5.2.6. Cefoperazone (see Table 4)

Varghese *et al.* [56] compared the kinetic parameters of cefoperazone after intramuscular and intravenous administration. The two administrations differ for the peak concentration that is higher after intravenous than intramuscular administration after a dose of 25 mg/kg cefoperazone. After a dose of 12.5 mg/kg cefoperazone there is no difference in the peak concentration between intramuscular and intravenous administration.

Bosso *et al.* [57] observed that the gestational age correlates with Cl (r *=* 0.67; p *=* 0.01) and with constant of elimination (r *=* 0.57; p < 0.05). t_1/2_ decreased with advancing gestational age (r *=* −0.81; p < 0.001; [58]). Vd ranged from 0.11 to 0.45 L/kg and t_1/2_ ranged from 3.9 to 8.9 h.

Rosenfeld *et al.* [59] compared the kinetic parameters of cefoperazone in preterm and term infants after a dose of 50 mg/kg. The only parameter that differs in the two development stages is the peak concentration which is greater in preterm than in term neonates. Vd ranged from 0.11 and 0.45 L/kg and t_1/2_ ranged between 2.8 and 8.9 h.

#### 5.2.7. Ceftizoxime (see Table 4)

The pharmacokinetics of ceftizoxime were studied in 52 infants whose postnatal age ranged from 1 to 189 days [60]. t_1/2_ diminished steadily as the postnatal aged increased whereas Cl had an opposite trend. Vd remained relatively constant. Ceftizoxime is excreted unchanged essentially by renal route [60]. Ceftizoxime Cl and Vd were strongly influenced by the body weight whereas the influence of gestational age on Cl and Vd was negligible [61]. Cl ranged from 0.68 to 28.5 mL/min/kg, Vd ranged from 0.32 to 0.44 L/kg, and t_1/2_ ranged between 2.4 and 9.7 h.

Young and Mangum [8] suggested administrating 50 mg ceftriaxone once daily. For treatment of meningitis, administrate a loading dose of 100 mg/kg followed by a daily dose of 80 mg/kg ceftriaxone.

### 5.3. Fourth generation cephalosporins

#### 5.3.1. Cefepime (see Table 4)

The serum creatinine concentration negatively correlates (r *=* -0.79) with cefepime Cl in neonates [62]. The serum concentration of creatinine is a strong predictor of cefepime Cl and the relationship between serum creatinine and Cl is similar across the range of gestational ages [62]. The relationship between cefepime Cl and gestational age is not significant. The development of the renal excretory function is an important determinant of cephalosporin dosing, including cefepime, in the neonate. In the premature, the renal function is impaired. Since cefepime is mainly excreted unchanged [62], the premature and term neonates clear cefepime more slowly than more mature infants. In neonates, cefepime Cl is about 40% of that in more mature infants, which results in a longer t_1/2_ and higher trough concentration in neonates. Vd is larger in infants less than 30 weeks of postconceptional life than in term neonates [62]. This is consistent with the larger total body water in the extremely premature neonate.

Reed *et al.* [63] described the pharmacokinetics of cefepime in 37 infants and children aged between 2 months and 16 years. The data are grouped on age; the youngest patients ranged between 2 and 6 months of age and the pharmacokinetic results of these patients are reported in Table 4. Ninety percent of cefepime was recovered in the urine over 24 h of urine collection; thus the elimination of cefepime is in large part via the kidneys. The data relative to cefepime revealed disposition parameters similar to those of third-generation cephalosporins, including linearity over a broad dose range (250–2000 mg), limited disposition and Cl mainly by the kidneys.

Lima-Rogel *et al.* [64] compared their own results on the pharmacokinetics of cefepime in neonates with those by Capparelli *et al.* [62] and with those by Reed *et al.* [63]. The pharmacokinetic parameters by Lima-Rogel *et al.* [64] and those by Capparelli *et al.* [52] were obtained in infants with similar demographic data t_1/2_ and Cl are comparable in these two studies. Reed *et al.* [63] described the pharmacokinetics of cefepime in older infants and children and in this study, t_1/2_ was one half and Cl was double those in the neonates. Cl ranged from 1.1 and 2.7 mL/min/mg, Vd ranged between 0.41 and 0.43 L/kg and t_1/2_ ranged from 1.9 to 4.9 h. 

The findings more relevant from a clinical point of view are as follows. A feature common to cephalosporins is the remarkable interindividual variability of their kinetic parameters. Such a variability is due to renal maturation as cephalosporin are fairly water soluble and are eliminated with the urine. t_1/2_ of cefotaxime, of ceftazidime and of ceftizoxime decrease with gestational and postnatal ages. t_1/2_ of cephalosporins ranged over a wide interval and the highest value (14.2 h) was observed for ceftazidime after intramuscular administration. Cephalosporins are safe, clinically effective and easy to use and the first and second generation are mostly used to treat infection sustained by Gram-positive bacteria, whereas the cephalosporins of the third and fourth generation are active against Gram-positive and several Gram-negative bacteria.

## 6. Aminoglycosides

**Table 5:** Number of retrieved studies and number of drugs evaluated.

Drug	Number of retrieved studies	Number of drugs evaluated	Overall number of neonates studied
Gentamicin	12	1	792
Netilmicin	10	1	510
Tobramycin	3	1	22
Amikacin	7	1	369
Total	32	4	1693

### 6.1. Gentamicin (see Table 6)

Aminoglycosides are toxic for the eighth cranial nerve [65,66] and for the kidney [65,67]. This requires the concentration of aminoglycosides to be within the appropriate interval. Gentamicin trough concentration >2 µg/mL is associated with toxicity [65,70] and peak concentration <5 µg/mL is associated with lesser efficacy [65,69] as gentamicin, as well as the other aminoglycosides, exhibits a concentration-depended bactericidal effect [65]. Formerly, gentamicin was administered at a dose of 2.5 mg/kg every 12 h [70]. Later, it appeared that once-daily gentamicin dosing of 4–5 mg/kg yields higher peak and lower trough gentamicin concentration than twice-daily dosing [for review see Rao *et al.* [71] and Miron [72]. Recently, administering 5 mg/kg gentamicin and extending the dose interval to 36-48 h has been recommended [73,74,75,76,77,78]. Extending the dose interval to 48 h and increasing the gentamicin dose to 5 mg/kg causes an increase in peak concentration as compared with the dose of 2.5 mg/kg every 12 h [74,75,76,77,78]. Begg *et al.* [77] observed that the optimal interval between doses is 48 h, 36 h, and 24 h for neonates whose body weight is <1 kg, 1–2.49 kg and ≥2.5 kg, respectively. The extended-interval method of aminoglycosides has been used in 75% of US hospitals since 2002 [79,80]. Cl ranged from 0.53 to 0.93 mL/min/mg, Vd ranged between 0.46 and 0.76 L/kg, and t_1/2_ ranged from 5.5 to 12 h.

Young and Mangum [8] suggested a gentamicin dose of 5 mg/kg every 48 h during the first week of life, when the gestational age is ≤29 weeks, a dose of 4.5 mg/kg every 36 h, during the first week of life, when the gestational age is 30 to 34 h and a daily dose of 4 mg/kg when the gestational age is ≥35 weeks.

**Table 6:** Pharmacokinetic parameters of aminoglycosides. Figures are the mean or range.

Gestational age (weeks)	Daily dose (mg/kg)	Cl (mL/min/kg)	Vd (L/kg)	t_1/2_ (h)	Peak conc. (µg/mL)	Trough conc. (µg/mL)	Ref.
**Gentamicin**							
26–32	2.5 every 18 h	0.93 mL/min	na	12.0	6.3	1.6	[70]
26	2.5	0.53	0.50	10.2	5.9	1.3	[81]
31	3.0	0.62	0.49	8.9	6.8	1.2	
38	4.0	0.78	0.46	7.0	8.9	1.3	
34	2.5 × 2	na	na	na	3.8	2.8	[82]
34	4	na	na	na	5.9	1.6	
p	---	---	---	---	<0.001	<0.05	
36	2.5 × 2	na	na	na	6.4	2.2	[83]
35	5	na	na	na	9.5	1.4	
p	---	---	---	---	<0.001	<0.005	
37–41	2.5 × 2	na	na	5.9	6.4	1.9	[84]
37–41	4	na	na	5.5	8.2	0.9	
p	---	---	---	na	<0.001	<0.0001	
29	2.5	na	0.76	11.1	6.0	1.25	[73]
28	5 every 48 h	na	0.76	10.7	8.1	1.72	
p	---	---	NS	NS	<0.0001	<0.001	
27	2.5	na	0.61	10.0	5.8	1.2	[74]
27	5 every 48 h	na	0.66	10.3	8.0	0.7	
p	---	---	NS	NS	<0.01	NS	
**Netilmicin**							
<34	4.5	1.5 (mL/min)	0.46	7.6	6.7	1.2	[85]
34–36	4.5	1.33 (mL/min)	0.51	8.5	8.6	1.4	
>36	4.5	3.16 (mL/min)	0.51	6.1	7.2	0.9	
29	6	0.45	0.52	6.6	5.0	2.7	[86]
35	6	0.45	0.46	6.7	6.2	2.8	
40	6	0.64	0.41	4.6	6.6	1.9	
35	3 (IM)	na	0.61	4.7	5.6	2.7	[87]
28	6 every 36 h	na	na	17.8	10	4.8	[88]
30	2.5 × 2	0.84	0.60	8.6	9.0	2.8	[89]
35	2.5 × 2 (IM)	1.06	0.34	9.6	7.7	2.6	[90]
**Tobramycin**							
28	Note A	0.69	0.59	9.9	7.6	1.7	[92]
29	Note B	0.72	0.74–0.94	8.3–12.8	4.6–8.4	1.2–2.0	[93]
na	5 (IM)	4.9 (mL/min)	0.49	4.4	2.7	na	[94]
**Amikacin**							
36	7.5 (IM)	1.42	0.66	6.0	18.6	na	[97]
32	5 (IM)	0.96	0.72	9.0*	na	na	[98]
40	1.24	0.56	0.55	5.5*	na	na	
34	5 (IM)	0.86	0.49	6.8	na	na	[99]
30	Note A	0.84	0.57	8.4	23.9	8.3	[100]
37 ^a^	Note B	2.05	0.34	2.8	6.8–35.7	<0.8–17.7	[101]
38	7.5–10	1.71	0.64	3.7	na	na	[102]
27^b^	Note E	0.36 ^d^	0.63 ^d^	16.4 ^d^	47.7 ^d^	9.9 ^d^	[105]
28 ^c^	Note E	0.60 ^d^	0.59 ^d^	12.4 ^d^	40.9 ^d^	6.2 ^d^	
p	NS	<0.005	NS	<0.02	NS	<0.01	

na *=* not available. NS *=* not significant. IM *=* intramuscular. ^a^ The postnatal age was 450 days and the body weight was 7.4 kg. ^b^ Ibuprofen. ^c^ Placebo. ^d^ Median. * p *=* 0.009. Note A: 2.5 mg/kg every 18 h or 3 mg/kg every 24 h. Note B: 2.5 mg/kg every 12 or 18 h. Note C: 7.5 mg/kg every 12 h when the chronologic life was up to 7 days and 7.5 mg/kg every 8 h when the chronologic life was >7 days. Note D: the loading dose was 11.7 ± 1.3 and the maintenance dose was 9.8 ± 1.4 mg/kg. In the first drug dosing regimen, neonates received a loading dose of 10 mg/kg amikacin followed by a maintenance dose of 7.5 mg/kg every 12 h. The infants and children received 7.5 mg/kg amikacin every 12 h. In the second drug dosing regimen, we considered an infusion of 15 mg/kg amikacin every 12 h for neonates, infants and children. Note E: 20 mg/kg every 36 h when gestational age was <30 weeks and 20 mg/kg every 24 h when gestational age was ≥30 weeks.

### 6.2. Netilmicin (see Table 6)

Table 6 shows the pharmacokinetic parameters of netilmicin at different gestation ages, different dosages and different administration routes. With increasing the gestational age from <34 to >36, t_1/2_ decreases from 7.6 to 6.1 h [85]. This is due to the development of the renal excretory function, as gentamicin, as well as the other aminoglycosides, is mainly eliminated by renal route. Administering 4.5 mg/kg netilmicin, Gosden *et al.* [85] have observed that the netilmicin peak concentration, which is expected to be >5 and <12 µg/mL and the trough concentration that should be <2 µg/mL, were within the expected values. Ettlinger *et al.* [86] made similar observations; when the gestational age increased from 29 to 40 week, t_1/2_ decreased from 6.6 to 4.6 h and the trough concentration decreased from 2.7 to 1.9 µg/mL. In this study, the trough concentration was >2 µg/mL in one third of the cohort. It must be noted that the trough concentration was measured 6 h after administration. The kinetic parameters of netilmicin are similar after intravenous and intramuscular administration [87]. The only information on the extended-interval administration of netilmicin was reported by Klingenberg *et al.* [87]. These authors administerd 6 mg/kg netilmicin every 36 h and report a very long t_1/2_ of 17.8 h; Cl is not available and the peak and trough concentrations are higher than those obtained after dosing of 6 mg/kg netilmicin once-daily [86]. Klingenberg *et al.* [88] suggested a dosing interval of 48 h for gestational age < 29 weeks, 36 h for gestational age ranging from 29–36 h, and 24 h for full term infants. Kuhn *et al.* [89] and Granati *et al.* [90] observed that the trough concentration was >2 µg/mL after administering 2.5 mg/kg twice daily. This suggests that once daily dose of 4 or 4.5 mg/kg netilmicin is safer than 2.5 mg/kg twice-daily. Recently, Sherwin *et al.* [91] proposed the following dosages of netilmicin 5 mg/kg ever 36 h, 6 mg every 24 h and 7 mg/kg every 24 h for neonates ≤27, 28–30 and ≥34 weeks of postmenstrual age, respectively.

Cl ranged from 0.45 to 1.06 mL/min/mg, Vd ranged between 0.34 and 0.61 L/kg, and t_1/2_ ranged from 4.6 to 17.8 h. Young and Mangum [8] suggested a dose of netilmicin similar to that of gentamicin given above.

### 6.3. Tobramycin (see Table 6)

The information on the pharmacokinetics of tobramycin in the neonate is limited. Nahata *et al.* [92,93] reported two articles that were based on premature neonates. Peak concentration ranged between 4.6 and 8.4 µg/mL and the trough concentration ranged between 1.2 and 2.0 µg/mL. Yoshioka *et al.* [94] administered 5 mg/kg tobramycin to neonates whose gestational age is unknown and the peak concentration was 2.7 µg/mL. de Hoog *et al.* [95] administered 4 mg/kg to all patients and the interval between doses was 48 h (<32 weeks), 36 h (32–36 weeks) and 24 h (≥37 weeks). Using this dosage schedule the majority of infants had peak concentration from 5 to 10 µg/mL and the trough concentration ranged from 0.5 and 1 µg/mL, then within the expected values. 

Nonlinear mixed effects model (NONMEM) and nonparametric expectation maximization (NPEM2) were used to estimate population parameters of tobramycin kinetics in neonates [96]. NONMEM showed less bias (p < 0.05) than NPEN2. Using NPEN2 Ke and Vd were 0.0079 h^−1^ and 0.65 L/kg, respectively. Young and Mangum [8] suggested a dose of tobramycin similar to that of gentamicin given above.

### 6.4. Amikacin (see Table 6)

Little is known on the pharmacokinetics of amikacin in the neonate. A study on the extended interval dosing of amikacin is lacking. Once-daily dosing of amikacin was administered by several authors [99]. Cl ranged between 0.56 and 1.42 mL/min/kg, Vd ranged from 0.49 to 0.72 L/kg and t_1/2_ ranged from 5.5 to 9.0 h. Kenyon *et al.* [10] studied the pharmacokinetics of amikacin after twice-daily administration. Cl, Vd and t_1/2_ were similar to those after once-daily dosing. These authors also measured the peak and trough concentrations that were 23.9 and 8.3 µg/mL. Tréluyer *et al.* [101] studied the pharmacokinetics of amikacin in infants with a mean postnatal age and body weight of 450 days and 7.4 kg, respectively. Cl was considerably higher than those found in neonates and t_1/2_ was shorter than those of neonates (Table 6). Wang *et al.* [102] studied the population pharmacokinetics of amikacin in 30 neonates with a mean gestational age of 38 weeks. These authors reported a Cl of 1.71 mL/min/kg and t_1/2_ of 3.7 L/kg and these values considerably differ from those obtained in the previous studies, see table 6.

Langhendries *et al.* [103] measured the peak and trough concentration of amikacin after once-daily and twice-daily dosing. After 15 mg/kg once-daily the peak was 23.1 µg/mL and, and after 7.5 × 2 it was 13.6 µg/mL. The trough concentrations were similar with two dosages. These authors suggested administering 15 mg/kg once-daily as the peak concentration obtained after 7.5 × 2 dosing is too low. Sherwing *et al.* [104] recently suggested the following dosages of amikacin 15 mg/kg every 36 h when the gestational age is ≤28 weeks, 14 mg/kg once-daily when the gestational age ranged from 29 to 26 weeks and 15 mg/kg once-daily when the gestational age is ≥37 weeks. Cl ranged from 0.36 to 2.05 mL/min/kg, Vd ranged between 0.49 and 0.72 L/kg, and t_1/2_ ranged from 2.8 to 16.4 h. 

Young and Mangum [8] suggested an amikacin dose of 18 mg/kg every 48 h during the first week of life when the postmenstrual age was ≤29 weeks; a dose of 18 mg/kg every 36 h during the first week of life when the postmenstrual age was 30-34 weeks; and a daily dose of 15 mg/kg when the postmenstrual age was ≥35 weeks.

The finding more relevant from a clinical point of view are reported hereafter. Aminoglycosides have a low therapeutic index and are toxic to the kidneyd and the acoustic nerve. To avoid toxicity, the peak and trough concentrations should be kept within the appropriate values which are < 12 and <2 µg/mL, respectively, for gentamicin, netilmicin and tobramycin, and <40 and <6 µg/mL, respectively, for amikacin. For the therapeutic doses see the text and a useful source of information is obtainable from Young and Mangum [8]. Aminoglycosides are used for the treatment of neonates with Gram-negative bacterial infection, which is potentially life-threatening. Aminoglycosides are administered in association with a penicillin, such as ampicillin or amoxicillin, as penicillins increase bacterial permeability to aminoglycosides. The high morbidity and mortality of bacterial infection in neonates require that the antibiotic therapy should be started as soon as the infection is suspected. 

## 7. Discussion

It is surprising that there have been few studies on the pharmacokinetics of penicillins, cephalosporins and aminoglycosides although they are key components of drug therapy in neonatal care units. The antibiotics that are more extensively studied are amoxicillin [16,17,18,19,20,21], cefotaxime [34,35,36], ceftazidime [41,42,43,44,45,46], gentamicin [70,73,74,75,76,77,81,82,83,84,106] and netilmicin [85,86,87,88,89,90,104] 

Most of the studies on the pharmacokinetics of penicillins, cephalosporins and aminoglycosides are classical pharmacokinetic studies where several blood samples were taken from each patient. Nevertheless, few studies of population pharmacokinetics have been provided for benzylpenicillin [11], for amoxicillin [16,18,21], flucloxacillin [22], for gentamicin [75,76,77,78,79,107], for netilmicin [104] and for amikacin [100,101]. The results of the population pharmacokinetic studies are consistent with those of the classical pharmacokinetic investigations.

Among penicillins, cephalosporins and aminoglycosides, this latter antibioticds are the most toxic, being toxic for the eighth cranial nerve [65,66] and the kidneys [65,67,68]. This requires that the concentration of aminoglycosides range within the appropriate values. The peak concentration of gentamicin should be >5 µg/mL and <12 µg/mL and the trough concentration should be <2 µg/mL [8]. This consideration also applies to netilmicin and tobramycin [8]. For amikacin the peak and the trough concentrations should be lower than 40 and 6 µg/mL, respectively [8]. The dose and the interval between doses of aminoglycosides have changed in neonates during recent years. Initially, the dose of gentamicin was 2.5 mg/kg every 12 h [70]. This dosage yields peak concentration <5 µg/kg and trough concentration >2 µg/mL in some patients. Increasing the gentamicin dose to 4–5 µg/mL once-daily, the peak and trough concentration of gentamicin ranged between 5 and 12 µg/mL. Increasing the gentamicin dose to 5 µg/mL and extending the interval between doses to 36–48 h the gentamicin peak and trough concentrations ranged between <12 and <2 µg/mL, respectively [75,76,77,78,79]. This consideration also applies to tobramycin and netilmicin. 

Gestational age and postnatal age have an important effect on Cl and consequently on t_1/2_ of penicillins [10,14,15,18,20,22,23,29], cephalosporins [33,38,42,43,45,57,60,63] and aminoglycosides [78,79,85,86,107]. Thus, gestational and postnatal ages are factors generating interindividual variability in the pharmacokinetics of penicillins, cephalosporins and aminoglycosides. Other factors that contribute to the interindividual variability in the pharmacokinetics of these antibiotics are malnutrition, disease and genetics. These factors coexist, and it is difficult to distinguish them among the different factors. Monitoring antibiotic serum concentration, particularly aminoglycosides, is necessary, especially in critical patients such as the preterm infants. Penicillins, cephalosporins and aminoglycosides are fairly water soluble and are mainly eliminated by renal route. The renal excretory function increases with gestational age as the GFR is lower in the premature than in term neonates [98,108] and increases with the postnatal age [46,49]. Cl of cefotaxime is 2–3 folds higher in term than preterm infants [34,35]. Gentamicin Cl correlates (r *=* 0.99) with creatinine Cl [109] and gentamicin t_1/2_ correlates (r *=* 0.78) with plasma creatinine concentration [106]. Cl of ceftazidime correlated with the reciprocal of the serum concentration of creatinine (r *=* 0.72; p < 0.001) suggesting that creatinine can interfere with the renal clearance of ceftazidime.

From a pharmacokinetic point of view, penicillins, cephalosporins and aminoglycosides are rapidly eliminated and their t_1/2_ ranges from 1.4 to 6.7 h for penicillins and to 16.2 h for ceftriaxone. In the preterm, t_1/2_ is longer than in full-term infants as these antibiotics are mainly eliminated by renal route and the excretory renal function increases with prenatal and postnatal development. This has been observed for azlocillin [24], ticarcillin [29], cefotaxime [35], ceftazidime [42], cefoperazone [59], gentamicin [81] and amikacin [98]. Cl has a reverse trend, it is lower in preterm than in full-term infants.

The present article is a comprehensive and updated review and summarises the pharmacokinetics of penicillins, cephalosporins and aminoglycosides in neonates in one article making it a useful tool in the hands of physicians. Paap and Nahata published a review on the clinical pharmacokinetics of antibacterial drugs in neonates in 1990 [7]. Although this article is a comprehensive review, the rapid progress of the pharmacology soon made it obsolete. In 1995, Lipuma *et al.* [110] published a review on the antibacterial agents in paediatrics, and in the same year, Sato [101] published an article on the pharmacokinetics of antibiotics in neonates. Recently, two reviews focused only on the pharmacokinetics of penicillins [112] or aminoglycosides [113] in neonates, and at present, there is not an updated review on the pharmacokinetics of cephalosporins in the neonate. The previous reviews were descriptive and do not give detailed information on the kinetic parameters of the various antibiotics. The present review adds in comparison with previous reviews an analysis of the kinetic parameters for penicillins, cephalosporins and aminoglycosides in the neonate. In addition, this review provides an analysis of the kinetics of cephalosporins in the neonate filling a space in the literature. 

Several factors influence the pharmacokinetics of penicillins, cephalosporins and aminoglycosides in the neonates. It is difficult to predict the pharmacokinetic behaviour of these drugs as many factors influence their disposition. The pharmacokinetics of these antibiotics should be investigated in order to ensure that the doses recommended for the treatment of sepsis are evidence based. 
